# Glycaemic Impact of Low‐ and High‐Glycaemic Index Carbohydrate Diets in Ultra‐Endurance Athletes: Insights From Continuous Glucose Monitoring

**DOI:** 10.1002/ejsc.70092

**Published:** 2025-11-25

**Authors:** Ross A. Hamilton, Ruiyang Xia, Chloe Nicholas, Rachel Churm, Olivia M. McCarthy, Richard M. Bracken

**Affiliations:** ^1^ Applied Sport, Technology Faculty of Science and Engineering Exercise and Medicine Research Centre Swansea University Swansea UK; ^2^ Copenhagen University Hospital Steno Diabetes Center Copenhagen Herlev Denmark; ^3^ Faculty of Science and Engineering Health Technology and Solutions Interdisciplinary Research Institute Swansea University Swansea UK

**Keywords:** endurance, metabolism, nutrition, performance, physiology

## Abstract

Nine ultra‐endurance athletes completed a randomised, crossover trial involving two 28‐day dietary arms during which the athletes consumed a carbohydrate‐rich diet (carbohydrate 58 ± 3, protein 15 ± 2 and fat 26 ± 2%) containing low‐ or high‐glycaemic‐index (LGI or HGI, respectively) carbohydrates. At the start and end of each dietary arm, participants performed a fasted 3‐h submaximal run outdoors before ingesting either a low (GI = 32, isomaltulose [Palatinose]) or high (GI = 100, maltodextrin) glycaemic index drink (0.75 g/kg bm/h over 3.5 h). Participants then completed a treadmill run to exhaustion at 74 ± 1% v$\dot{V}$O_2peak_, with pulmonary gas exchange measured over the first hour. Interstitial glucose [iG] was measured via continuous glucose monitoring (Supersapiens, Atlanta, USA). Data were analysed ANOVA and post hoc *t*‐tests with Bonferroni adjustment as appropriate, with *p* ≤ 0.05 accepted as significant. Mean 24‐h [iG] was similar between diets (LGI:102 ± 5 vs. HGI:100 ± 5 mg/dL). [iG] variability measures, including standard deviation (LGI:17 ± 1 vs. HGI:18 ± 2 mg/dL, *p* = 0.016) and coefficient of variation (LGI:16 ± 1% vs. HGI:18 ± 1%, *p* = 0.0003), were lower in the LGI diet, with a reduced percentage of time spent below the recommended range (LGI 2 ± 1% vs. HGI 4 ± 2%, *p* = 0.006. Level 1 [55–69 mg/dL] LGI 1 ± 1% vs. HGI 3 ± 2, *p* = 0.005). Carbohydrate oxidation during the first hour of the run test was reduced in the LGI diet arm (ΔLGI −0.14 ± 0.32 vs. ΔHGI 0.06 ± 0.28 g·min^−1^, *p* = 0.016) but endurance capacity was similar across diets. Adopting a 28‐day LGI carbohydrate‐rich diet and incorporating isomaltulose improved glycaemic variability and reduced time spent below the target glycaemic range with evidence of similar endurance performance capability when compared to a HGI carbohydrate‐rich diet.

## Highlights

- This study provides a detailed glycaemic profile of healthy athletic individuals over a prolonged period of heavy endurance exercise following a nutrition intervention.
- Adopting a 28‐day LGI carbohydrate‐rich diet improved glycaemic variability and reduced time spent below the euglycaemic range when compared to a HGI carbohydrate‐rich diet.
- Endurance capacity was maintained equally under both diet arms.

## 1 Introduction

In recent years, there has been a rise in the popularity of ultra‐endurance athletic events. Ultra‐endurance events can exceed 6 h and place large demands on body stores of energy for athletes to perform successfully. Recommendations for carbohydrate (CHO) consumption for endurance activities suggest dietary intake of > 60% of total daily energy or between 8 and 12 g·kg^−1^ day^−1^ for competition (T. Thomas et al. 2016). Further, in training, ultra‐endurance athletes complete large weekly volumes of exercise activities (Rüst et al. 2012; Tanda and Knechtle 2015), and to meet such high energetic demands, carbohydrates form the mainstay of daily energy intake (Stellingwerff 2016). Exogenous carbohydrate intake substantially impacts blood glucose concentrations, and if large amounts and/or high glycaemic index (HGI) sources are consumed, dysglycaemia, characterised by pronounced rises and falls from an otherwise stable glucose level, can occur despite endogenous gluco‐regulatory mechanisms striving to maintain glucose homeostasis (Bazzano et al. 2005).

Recent technological developments have led to the emergence of continuous glucose monitors (CGMs) to manage glycaemia in metabolically dysregulated populations (Galindo and Aleppo 2020). Continuous real‐time recording and display provide a 24‐h continuous trajectory of glucose around meals, physical activity and during sleep (Shah et al. 2019; Keshet et al. 2023). During competition, maintaining stable interstitial glucose levels has been suggested to positively impact performance outcomes, such as sustaining running pace (Sengoku et al. 2015; Ishihara et al. 2020). However, the implications of day‐to‐day glucose variability on exercise performance is not currently well understood.

Longer studies, have profiled glycaemia over short periods (< 19 days) of intensified training (Francois et al. 2018; Zignoli et al. 2023; Bowler et al. 2024; Hamilton et al. 2024; Skroce et al. 2024). These studies characterised glycaemia using a variety of different metrics such as the percentage of time spent in different ranges (hyper‐, eu‐ or hypo‐glycaemia), albeit with varying threshold concentrations, and frequency and severity of hypo‐ and hyperglycaemia. These initial investigations have detailed observations of dysglycaemia in athletes (T. Thomas et al. 2016; Francois et al. 2018; Kulawiec and et al. 2021; Flockhart and Larsen 2023; Hamilton et al. 2024). Furthermore, during periods of heavy training, Flockhart et al. (2021) reported significantly greater time spent above range in athletes compared to a healthy control group. Studies have observed some modes TAR in healthy athletic cohorts (Bowler et al. 2024; Hamilton et al. 2024; Weijer et al. 2024; Zignoli et al. 2024). In addition, frequent periods of hypoglycaemia were also observed, although often specific to the nighttime period. Interestingly, studies have also used mean, median, standard deviation, coefficient of variation and mean amplitude of glycaemic excursion (MAGE) to explore glycaemic characteristics over time.

Few studies have observed glycaemia in athletic cohorts for more prolonged periods, with very few detailing glycaemia in response to chronic nutritional interventions. Longer periods of CGM utilisation appear to have a greater benefit to glycaemic management (Anderson et al. 2011). With that in mind, longer study durations may be necessary to investigate some physiological adaptations. Further investigation is warranted to explore the metabolic and health impacts associated with glycaemic management in response to different sports nutrition strategies. Prins et al. (2023) demonstrated that 5 weeks of a low carbohydrate high fat (LCHF) diet reduced 24‐h mean glucose and resulted in greater fat oxidation during an exercise assessment at the end of the intervention, when compared to a high carbohydrate low fat diet. Other alternative strategies, such as isocaloric low glycaemic diets, might also alter glycaemia and performance outcomes, but have not been explored in athletes.

Consumption of low glycaemic index (LGI) carbohydrates typically results in a slower rise and lower peak glucose response that can aid stable glucose concentrations (Jenkins et al. 1981). As such, they might help prevent hypoglycaemia around exercise (Burke et al. 1998; Ching‐Lin et al. 2003). Pre‐exercise meals containing LGI carbohydrates are effective at maintaining exercise glycaemia, especially when exercise is prolonged and feeding opportunities are limited (D. Thomas et al. 1991; Wu and Williams 2006). LGI carbohydrates incorporated into pre‐exercise meals have shown improvements of 2.8%–3.3% in time‐trial performances when compared to meals comprised of HGI carbohydrate (Wong et al. 2008; Moore et al. 2009, 2010). Overall, the literature demonstrates equivalent, or in some cases, small improvements in performance in comparison to consuming isocaloric amounts of moderate or high GI carbohydrates (Burdon et al. 2017). One such low glycaemic index carbohydrate is isomaltulose (Palatinose) which is a disaccharide sucrose isomer of glucose and fructose; it has a hydrolysation rate that is 20%–25% of that of sucrose (Gunther and Heymann 1998; Lina et al. 2002), giving it a glycaemic index value of 32. As a result, meals and diets incorporating isomaltulose display lower glycaemic responses when compared to meals and diets incorporating higher GI carbohydrates (van Can et al. 2009; Henry et al. 2017; Maresch et al. 2017; Notbohm et al. 2021).

Some research studies have effectively used simple dietary alterations to improve glycaemia, observed via CGM (Bergia et al. 2022; Chekima et al. 2022) in healthy but nonathletic cohorts. With such emphasis on carbohydrate intake, and its importance for exercise performance, gaining better insights into how GI might impact the glycaemia of a highly active individual who consumes a carbohydrate‐rich diet for their athletic endeavours is of great interest, especially in the context of exercise performance. Thus, we hypothesise the chronic consumption of LGI carbohydrate‐rich diets might aid glycaemic stability in endurance athletes while maintaining carbohydrate provision for energy compared to conventional high glycaemic index diets, but this has not been researched nor has its impact on performance been explored.

Thus, this randomised, repeated crossover study examined the glycaemic impact of adopting a 28‐day carbohydrate‐rich diet, primarily consisting of either low‐ or high‐GI carbohydrates on acute and chronic glycaemia using continuous glucose monitoring, and explored the impact on endurance capacity in ultra‐endurance athletes.

## 2 Materials and Methods

### 2.1 Participants and Ethical Approval

Nine ultra‐endurance trained athletes (8 male) took part in this randomised, cross‐over study (age: 41 ± 7 years, height: 176 ± 9 cm, body mass: 79 ± 16 kg). Ethical approval was granted by the Swansea University Research Ethics Committee. The study was carried out in accordance with the Declaration of Helsinki and International Conference on Harmonisation of Good Clinical Practice. All volunteers provided written informed consent prior to study involvement.

### 2.2 Screening Visit

Before undertaking any experimental procedures, participants completed a screening visit during which eligibility for trial inclusion was assessed alongside a review of their medical history via the PAR‐Q questionnaire. After confirmation of study suitability (based on inclusion criteria and providing sufficient evidence of active participation in ultra‐endurance exercise), data on anthropometric characteristics were collected before participants completed a treadmill ramp test to volitional exhaustion to determine individualised intensity thresholds for subsequent experimental visits. After a 10‐min warm‐up, participants completed a 5‐min standing rest, followed by a 3‐min incremental treadmill test to volitional failure, with speed increasing by 1 km·h^−1^ per stage (BASES 2006). The treadmill gradient remained at 1%, and breath‐by‐breath data were recorded using a pulmonary gas analyser (MetaMax 3B; Cortex Biophysik GmbH, Germany). Blood samples were analysed using the Biosen C‐Line system (EKF Diagnostics). Participants were then familiarised to procedures by running a portion of the outdoor course and treadmill test at their assigned velocity. They were also instructed on how to use physical activity logging apps and CGM sensors (and ecosystem) to allow them to familiarise themselves to its use over the 7 days preceding the commencement of the study.

### 2.3 Study Design

**FIGURE 1:**
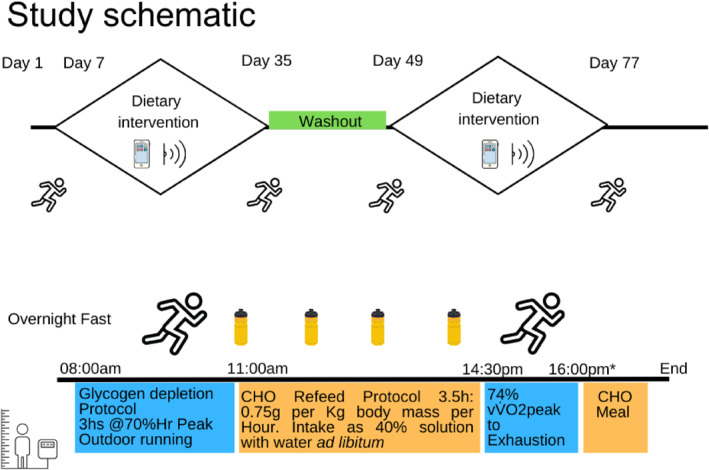
Study schematic. Study overview is displayed from Day 1 to Day 77. The laboratory exercise trials are indicated and continuous glucose monitor (CGM) collection with the phone and scanner symbol. The timeline of the acute laboratory days is also included in the lower portion of the figure providing indicative times for each stage of the trial. Anthropometry was recorded as soon as participants arrived (indicated by the figure and scale symbol) before they began their outdoor run at 71 ± 2% heart rate (HR) peak. They then completed the carbohydrate (CHO) refeed (0.75 g [g] per kilogram [kg] body mass), indicated by bottle symbols. Both the 3‐h outdoor run and treadmill test to exhaustion (at 74 ± 1% v$\dot{V}$O_2peak_) are indicated by running man symbols. * The exact finish time was dependent on the outcome of the run capacity test.

As part of the randomisation (via computerised, randomised sequence), participants were allocated to start with either LGI or HGI carbohydrate‐rich diets over a 28‐day period before crossing over to the opposing dietary arm. After an initial 7‐day regular habitual diet, the first 28‐day diet arm began the day after the initial acute laboratory visit, then ended on Day 35 (the second acute laboratory day of the arm) and the athlete then returned to their regular diet for a 14‐day washout period; athletes then began the second diet arm for another 28 days and finished on Day 77. At the start and end of each dietary arm, participants attended four laboratory arm days that involved the evaluation of acute LGI (isomaltulose) or HGI (maltodextrin) carbohydrate responses to endurance exercise capacity tests. A study schematic is displayed in Figure 1. Participants maintained their routine exercise training regimes throughout the 28‐day dietary periods, logging all exercise sessions via GPS sports watches uploading data to TrainingPeaks (TrainingPeaks, Peaksware LLC, Louisville, USA). This data was then accessible to the research team for retrospective analysis.

### 2.4 Experimental Trial Day Procedures

Participants attended the laboratory after an overnight fast (≥ 10 h) having avoided any physical activity in the preceding 24 h. After gathering anthropometric measures (height, body mass, estimated body fat and lean percentages via bioelectric impedance analysis) (Bodystat Quadscan 4000, Bodystat Ltd, USA), participants proceeded to run outdoors on a standardised pre‐measured course for 3 h at an intensity equivalent to 70% $\dot{V}$O_2peak,_ monitored via heart rate telemetry. HR was kept within ± 5 bpm of this pre‐determined intensity with run data collected from their own GPS watch, later downloaded from TrainingPeaks. Participants were encouraged to consume water with added electrolyte powder (Bulk, Chichester, United Kingdom) during this run.

Following the outdoor run, participants returned to the laboratory where they consumed a carbohydrate drink (0.75 g.kg^−1^ BM h^−1^ as 40% fluid solution [approx 2.7 g/kg BM]) containing 1 g of electrolyte powder with either (i) the LGI carbohydrate; isomaltulose (ISO; Palatinose) (BENEO, Mannheim, Germany) or (ii) the HGI carbohydrate; maltodextrin (MAL) (BENEO, Mannheim, Germany). Subsequent carbohydrate refeeding took place under rested conditions over 3.5 h. Thereafter, participants began an indoor treadmill test where they ran at an intensity equivalent to 74 ± 1% v $\dot{V}$O_2peak_ (11.65 ± 0.60 km.h^−1^) until volitional fatigue. Continuous measures of cardiopulmonary data were collected for the first hour of this test.

### 2.5 Collection of Glycaemic Data

All interstitial glucose [iG] data were recorded via the Abbot Librae Sense Biosensor (Abbot Laboratories, Chicago, IL, USA). The CGM device was paired to the SuperSapiens fuel band receiver and Software application (TT1 Products Inc., Atlanta, GA, USA) which was installed on the participant's smartphone. Raw CGM data were exported to a database and analysed via Excel 2019 (Microsoft Corp., Redmond, WA, USA).

Group means were calculated for [iG] concentrations (mg/dL) and indices of glycaemic variability, that is, the coefficient of variation (CV) and standard deviation (SD). [iG] data were also stratified into a percentage of time spent in specific glycaemic ranges: time below range ([TBR] < 70 mg/dL), time in range ([TIR] 70–140 mg/dL) and time above range ([TAR] > 140 mg/dL). Hypoglycaemia was further stratified into Level 1 hypoglycaemia ([LVL1] 55–69 mg/dL) and Level 2 hypoglycaemia ([LVL2] ≤ 54 mg/dL).

### 2.6 Collection of Dietary Information

After attending the screening visit, participants followed their regular diet for a period of 7 days, recording all dietary intake using the Nutritics smartphone application (Nutritics, Dublin, Ireland). Based on this information, they were advised on suitable substitutions to ensure they were made aware of either LGI or HGI variations of their preferred carbohydrate foods, for example, for a low GI swap, a white potato was exchanged for a sweet potato. They were provided with food lists detailing suitable food options for each trial arm based on their usual food choices.

In addition, over each 28 day diet and to further enhance the different glycaemic properties of each diet, participants supplemented their physically active lifestyle with low (isomaltulose) or high (maltodextrin) GI carbohydrate drinks, respectively. Participants were encouraged to follow dietary recommendations of 7 g.kg.d^−1^ of carbohydrates (T. Thomas et al. 2016). Around exercise activities, athletes were encouraged to consume each low‐ or high‐GI carbohydrate in solution (e.g., 50 g of either ISO or MAL in 550 mL of water as a 9% solution 1–2 h before exercise as well as every hour during exercise). In the first 90 min of recovery from an exercise session, participants were encouraged to consume 0.75 g.kg BM^−1^ of the low‐ or high‐GI carbohydrate with water. This was not only to encourage adequate carbohydrate intake but also to ensure the carbohydrate was appropriate to the dietary arm they were assigned to. All dietary intake information was continuously analysed for verification of adherence by the research team and continuous guidance was provided to ensure appropriate dietary choices were appropriate.

### 2.7 Collection of Training Information

Participants followed their own physical training programmes throughout each of the 28‐day diet arms. All training data were collected by the individual participants' GPS sports watches. Each participant's data was subsequently imported to the TrainingPeaks application, downloaded and sent to members of the research team for analysis.

For the retrospective classification of exercise intensities, a three‐zone training model was utilised. Intensity zones were defined with HR using the first and second lactate threshold turning points as identified by the lactate curve from the graded incremental exercise test (Seiler 2010). Assessments of overall duration and distribution of training intensity were retrospectively made after each diet.

### 2.8 Statistical Analyses

Statistical analyses were carried out using Excel (Microsoft Office) and Graphpad Prism V 9.5. All data are presented as mean ± standard deviation (SD). Data were tested for normal distribution (Shapiro–Wilk test) A one‐way ANOVA was conducted to compare differences between anthropometrics, exercise metrics, glycaemic metrics (day to day) and metabolic data. When significant main effects were identified, Bonferroni post hoc adjustments were applied to correct for multiple comparisons and identify where the differences were observed. A two‐way ANOVA was employed to assess the interaction effects between glycaemic metrics between trials and timepoints across the acute trial day. If significant interactions were found, simple main effects were analysed using Bonferroni‐corrected pairwise comparisons. Finally, for pairwise comparisons (LGI vs. HGI trail arm means), paired *t*‐tests were used. Significant differences were reported if *p* ≤ 0.05.

Sample size estimates indicated that the number of participants required to achieve adequate statistical power varied widely across glycaemic metrics, reflecting the differing magnitudes of responses. Given this variability, it was not feasible to recruit a sample large enough to achieve 0.80 power for all outcomes within the study timeframe. Post hoc effect size analysis supported this variability, showing effects ranging from trivial to very large. Although differences in mean and maximum glucose were small, measures of glycaemic variability (SD, CV) and time below range (TBR, LVL1) showed large to very large effects.

## 3 Results

### 3.1 28‐Day Glycaemic Data

#### 3.1.1 24‐h [iG]

The 28‐day glycaemic group 24 h mean [iG] variables are shown in Table 1.

**TABLE 1:** Mean interstitial glucose metrics over for both 28‐day low‐ (LGI) and high‐glycaemic index (HGI) carbohydrate rich diet arms.

	LGI carbohydrate diet	HGI carbohydrate diet	95% CI	*p* value
Max (mg/dL)	168.8 ± 9.2	169.9 ± 8.2	−3.528 to 5.732	*p = 0.598*
Mean (mg/dL)	101.6 ± 4.6	100.0 ± 5.0	−6.289 to 2.921	*p = 0.424*
Min (mg/dL)	63.6 ± 2.8	61.2 ± 1.6	−4.401 to −0.05152	***p = 0.046***
SD (mg/dL)	16.7 ± 1.7	18.3 ± 1.7	0.3821 to 2.726	***p = 0.016***
CV (%)	16 ± 1%	18 ± 1%	1.298 to 2.947	***p = 0.0003***
TAR (%)	4 ± 2%	4 ± 2%	−0.8634 to 1.386	*p = 0.607*
TIR (%)	93 ± 4%	91 ± 3%	−5.288 to 2.646	*p = 0.465*
TBR (%)	2 ± 1%	4 ± 2%	0.8570 to 3.559	***p = 0.006***
LVL1 (%)	1 ± 1%	3 ± 2%	0.6497 to 2.561	***p = 0.005***
LVL2 (%)	0 ± 0%	1 ± 1%	−0.1011 to 1.357	*p = 0.082*

Abbreviations: CI, Confidence limits; CV, Coefficient of variation; LVL1, The percentage of time spent with interstitial glucose levels within a range (55–69 mg/dL); LVL2, The percentage of time spent with interstitial glucose levels below the target range (≤ 54 mg/dL); Max, maximum concentrations of participants over each 28‐day period; Mean, average mean concentrations of participants over each 28‐day arm; Min, minimum concentrations of participants over each 28‐day period; SD, Standard deviation; TAR, The percentage of time spent with interstitial glucose levels above the target range (> 140 mg/dL); TBR, The percentage of time spent with interstitial glucose levels below the target range (< 70 mg/dL); TIR, The percentage of time spent with interstitial glucose levels within a target range (70–140 mg/dL).

*Note:* Data displayed as mean ± SD (*n* = 9). *p* values in bold italics indicate a significant difference (*p* ≤ 0.05).

### 3.2 Time in Glycaemic Ranges

Although the mean [iG] concentrations were similar during both 28‐day diet arms, measures of variance, that is, SD and CV, were lower in the LGI compared to the HGI diet arms (*p* = 0.016 and *p* = 0.0001, respectively). TBR (LVL1) was higher, and [iG] minimum lower, in the HGI diet arm (Table 1).

### 3.3 28‐Day Dietary Intake

Carbohydrate intake was ∼58% of total daily energy intake in both diets. The daily intake of supplemental carbohydrates using the pre‐formulated carbohydrate powders to daily meals was equivalent between diet arms, accounting for ∼30% of overall daily intake (LGI 32 ± 9 vs. HGI 28 ± 13%, *p* = 0.402). There were no differences in the overall energy intake (LGI 3044 ± 452 vs. HGI 2961 ± 233 kcals, *p* = 0.562) or the amounts of carbohydrate (LGI 443 ± 41 vs. HGI 429 ± 70 g, *p* = 0.486) and fat (85 ± 9 vs. HGI 83 ± 7 g, *p* = 0.283) consumed between dietary arms. However, more protein was consumed during the LGI diet arm (121 ± 15 vs. 107 ± 11g, *p* < 0.001).

### 3.4 28‐Day Physical Activity Data

The total exercise duration undertaken throughout the 28‐day was similar between the low‐ and high‐GI arms (LGI 37.9 ± 9.5 vs. HGI 34.5 ± 7.5 h, *p* = 0.165). Time spent in Z1 was similar (LGI 25.8 ± 11.9 vs. HGI 22.7 ± 9.5 h, *p* = 0.450), as was time spent in Z2 (LGI 6.4 ± 3.6 vs. HGI 6.5 ± 2.0 h, *p* = 0.997) and Z3 (LGI 1.4 ± 2.0 vs. HGI 1.1 ± 0.7 h, *p* = 0.700). There was no difference in the distribution of training intensities between the dietary arms.

### 3.5 Trial Day [iG]

#### 3.5.1 Trial Day Blood Analysis

Fasting blood glucose and serum insulin concentrations are displayed in Table 2. These remained similar between trials and were within normal fasting ranges. HOMA IR and QUICKI assessments were also performed, and both were in normal range and similar between trials.

**TABLE 2:** Displays fasted blood glucose and serum insulin concentrations.

	ISO pre‐diet	ISO post‐diet	MAL pre‐diet	MAL post‐diet	Overall *p* value
Fasted blood glucose (mmol/L–1)	4.6 ± 0.47	4.55 ± 0.56	4.23 ± 0.26	4.23 ± 0.30	*0.253*
Fasting serum insulin (μIU/mL)	8.6 ± 7.4	8.4 ± 5.1	8.0 ± 3.4	11.6 ± 8.1	*0.503*
HOMA IR	1.6 ± 1.6	1.5 ± 1.3	1.1 ± 0.9	2.0 ± 1.8	*0.452*
QUICKI	0.36 ± 0.04	0.37 ± 0.04	0.36 ± 0.03	0.36 ± 0.05	*0.69*

*Note:* In addition, Homoeostatic Model Assessment for Insulin Resistance (HOMA IR) and Quantitative Insulin Sensitivity Check Index (QUICKI) are presented. All data are displayed as mean ± SD. *p* values in bold italics indicate a significant difference (*p* ≤ 0.05).

**FIGURE 2:**
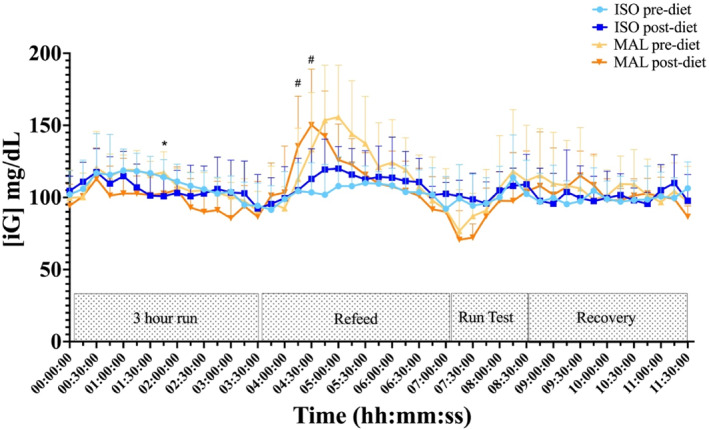
Mean interstitial glucose [iG] concentrations under each carbohydrate (isomaltulose [ISO] or maltodextrin [MAL]) condition before (pre) and after (post) each 28‐day diet arm. Time course of the laboratory trial day has been smoothed into 15‐min intervals. Run test: run test to exhaustion. * indicates a difference in the respective point concentration of [iG] between ISO arms before and after the 28‐day diet. # indicates a difference in the respective point concentration of [iG] between ISO and MAL arms after the 28‐day diet arm (*p* ≤ 0.05). Data are presented as mean ± SD (*n* = 9).

[iG] data during each acute trial day was predefined into time segments; 3 h run, refeed, run test and the recovery period. A continuous CGM trace for the acute trial day is displayed in Figure 2 below (Summary time‐segmented data are displayed in supplemental Table 1). Mean [iG] was similar before and after a run test to exhaustion whether at the start or end of a 28‐day low‐ or high‐GI carbohydrate diet. However, measures of variance, standard deviation (*p* = 0.001) and coefficient of variation (*p* = 0.002) were lower for both ISO arms compared to MAL during the carbohydrate refeeding period, in the performance run test to exhaustion (SD *p* = 0.05, CV *p* = 0.008) and in the subsequent recovery period (SD *p* = 0.01, CV *p* = 0.002).

#### 3.5.2 3h Standardised Run Data

The total distance covered over the standardised 3‐h run was similar between all acute trial arm days (ISO_pre_ 25.1 ± 2.3, ISO_post_ 27.0 ± 2.9, MAL_pre_ 25.3 ± 2.7, MAL_post_ 26.5 ± 2.7 km, *p* = 0.352), as was mean HR (ISO_pre_ 126 ± 6, ISO_post_ 126 ± 6, MAL_pre_ 129 ± 5, MAL_post_ 128 ± 6 bpm, *p* = 0.862) and speed (ISO_pre_ 8.4 ± 0.9, ISO_post_ 9.0 ± 1.0, MAL_pre_ 8.3 ± 1.0, MAL_post_ 8.6 ± 1.1 km.h^−1^, *p* = 0.445). Exercise intensity (expressed as percentage of HRmax) was also similar during the fasted morning run performed at the start of each trial day (ISO_pre_ 70 ± 2, ISO_post_ 71 ± 3 MAL_pre_ 70 ± 3, MAL_post_ 70 ± 4% HR_max_, *p* = 0.904).

#### 3.5.3 Endurance Capacity Run Test Data

Endurance capacity run test data are displayed in Table 3.

**TABLE 3:** Summary data for each time to exhaustion treadmill test during each acute trial day visit under each carbohydrate (isomaltulose [ISO] or maltodextrin [MAL]) condition both before (pre) and after (post) each 28‐day period, as well as the changes within.

	ISO _pre‐diet_	ISO _post‐diet_	MAL _pre‐diet_	MAL _post‐diet_	Overall *p* value	Δ LGI	Δ HGI	*LGI* versus *HGI*
Mean time to failure (mins)	50 ± 20	65 ± 15	69 ± 23	72 ± 16	*p = 0.104*	+15 ± 17	+5 ± 24	*p = 0.414*
Heart rate (bpm)	157 ± 6	158 ± 9	161 ± 11	157 ± 10	*p = 0.140*	−2 ± 6	−5 ± 6	***p = 0.014***
Percent of HR_max_ (%)	88 ± 5	87 ± 3	89 ± 4	86 ± 3	*p = 0.533*	−1 ± 3	−3 ± 3	*p = 0.270*
$\dot{V}$O_2_ (mL.kg^−1^.min^−1^)	42 ± 4	42 ± 3	41 ± 4	41 ± 5	*p = 0.932*	+1 ± 4	+5 ± 11	*p = 0.280*
RER	0.94 ± 0.02^a^	0.94 ± 0.01^b^	0.96 ± 0.02	0.97 ± 0.01	*p < 0.001*	+0.03 ± 0.07	−0.02 ± 0.02	*p = 0.152*
RPE (bourg)	15 ± 2	15 ± 1	15 ± 2	15 ± 2	*p = 0.772*	NA	NA	NA

*Note:* *p* values in bold italics indicate a significant difference (*p* ≤ 0.05) in the corresponding variable between the two dietary arms. Δ denotes the change within each of the dietary arms. All data are displayed as mean ± SD (*n* = 9).

Abbreviations: Time to exhaustion (min), heart (bpm), ratings perceived exertion, volume of oxygen uptake ($\dot{V}$O_2_ mL.kg^−1^.min^−1^) and respiratory exchange ratio (RER) are displayed.

^a^ indicates a difference between ISO and MAL before the 28‐day diet.

^b^ indicates a difference between ISO and MAL after each 28‐day diet.

#### 3.5.4 Fuel Oxidation Data During the Indoor Treadmill Run to Exhaustion

**FIGURE 3:**
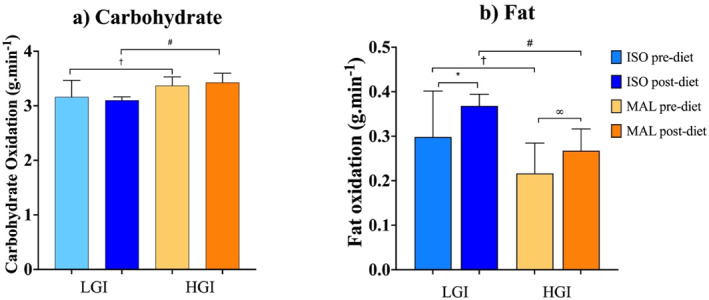
Summary of fuel oxidation rates for (a) carbohydrate and (b) fat in the first hour of the endurance capacity run test under both carbohydrate (isomaltulose [ISO] or maltodextrin [MAL]) conditions, both before (pre) and after (post) each 28‐day low‐glycaemic index (LGI) and high‐glycaemic index (HGI) diet arms, as well as the changes within. * indicates a difference between ISO pre and post 28 days. ∞ indicates a difference between MAL pre and post. † indicates a difference between ISO and MAL before the 28‐day diet. # indicates a difference between ISO and MAL after each 28‐day diet (*p* ≤ 0.05). All data are displayed as mean ± SD (*n* = 9).

Fuel oxidation data are displayed in Figure 3. There was a greater oxidation rate of lipids and lower oxidation of carbohydrates during the first hour of the indoor treadmill run to exhaustion with consumption of ISO compared to MAL both at the start and end of the 28‐day diet arms.

#### 3.5.5 Anthropometric Changes

Anthropometric measures: body mass, Est body fat, fat mass, Est LBM and LBM BMI (See, Table 4) remained similar across both trial arms (*p* < 0.05).

**TABLE 4:** Participant characteristics. LBM is lean body mass. All data are displayed as mean ± SD.

Age (years)	Height (cm)	Body mass (kg)	Est body fat (%)	Fat mass (kg)	Est LBM (%)	LBM (kg)	BMI (kg/m^2^)	$\dot{V}$O_2_ max (mL.kg^−1^.min^−1^)
41 ± 7	176 ± 9	79.8 ± 16	21.3 ± 5.4	17 ± 7.1	78.6 ± 5.4	62 ± 9.8	25.5 ± 3.3	56.9 ± 3.9

## 4 Discussion

This study demonstrated that the adoption of a carbohydrate‐rich diet consisting of low‐glycaemic index carbohydrates by ultra‐endurance athletes over 28 days reduced glycaemic variability and time spent below the target range compared to the high‐GI carbohydrate diet. Furthermore, low‐GI carbohydrate diets reduced carbohydrate oxidation during submaximal exercise, but had no impact on run capacity to exhaustion compared to the isoenergetic HGI carbohydrate diet.

### 4.1 Glycaemia

The average lowest interstitial glucose concentrations were higher by 3 ± 4% under the low‐GI carbohydrate‐rich diet compared to the high‐GI carbohydrate diet over the 28 days. In addition, in the LGI carbohydrate diet arm, we found a lower standard deviation (Δ LGI −1.55 ± 1.52 mg/dL, *p* = 0.016) and coefficient of variation (Δ LGI −2 ± 1%, *p* < 0.001) in interstitial glucose concentrations. These findings demonstrate a lower variation around the mean values and suggest daily rises and falls of circulating glucose were less in the LGI carbohydrate arm compared with the HGI carbohydrate arm. Other studies have also reported other lower measures of variance with LGI carbohydrate diets compared to high glycaemic alternatives, for example, mean amplitude of glycaemic excursion (MAGE) (Henry et al. 2017; Kaur et al. 2016). MAGE measures glucose variability by averaging differences between peaks and nadirs, considering only excursions exceeding one standard deviation. Its accuracy relies on consistent, frequent data recording. Irregular intervals, data gaps and multiple CGM inputs can skew estimates. In our study, we obtained raw data from files, not app‐generated findings, so some signal breaks introduced gaps and variability, and therefore, MAGE was not able to be assessed.

Though it might be surmised that the HGI carbohydrate‐rich diet raises circulating glucose more than an LGI carbohydrate diet, we did not see this in our data of maximum values. Participants displayed similar average daily and mean interstitial glucose concentrations over each 28‐day arm while on the LGI or HGI carbohydrate diets. A 24‐h ‘time in tight range’ (TITR) principle was applied to assess glycaemia in this study. TITR is a relatively recent progression from the standard TIR and it is the model utilised by the SuperSapiens software package. TITR sets a tighter band of concentration (70–140 mg/dL [3.9–7.8 mM]) than current clinical recommendations (70–180 mg/dL [3.9–10 mM]). The time spent above, in or below standardised glucose range in people without diabetes, provides a useful way of detailing the percentage of time spent in the ‘extremes’ of high or low glucose concentrations. Our data showed that when participants were on the LGI carbohydrate‐rich diet, the average time spent below 70 mg/dL each day over 28 days was half of that observed in the HGI carbohydrate diet arm. Our data suggest that LGI carbohydrate diets that incorporate isomaltulose may result in less time spent in hypoglycaemia (TBR) in ultra‐endurance athletes.

The clinical significance of Level 1 hypoglycaemia in healthy individuals is somewhat debatable, with the suggestion that values below 70 mg/dL (3.9 mM) are more an indication of low glucose rather than of clinical concern (Danne et al. 2017). These values are somewhat arbitrarily set, as symptoms of hypoglycaemia can begin to occur at a wider range of lowered blood glucose concentration and are highly individualised (Jeukendrup and Killer 2010; Simpson et al. 2008). However, Level 1 hypoglycaemia marks an alert value for corrective intervention for those with type 1 diabetes for obvious reasons. Exposure to glucose at this lowered level may not induce overt physiological symptoms but falls below it may initiate milder symptoms such as sweating, shaking and hunger in some individuals (Cryer 2007). For athletes, there is a risk of impaired cognitive and physical performance (Brun et al. 2001). In this study, TBR was spent in Level 1 hypoglycaemia, although some Level 2 was experienced under both diets (∼1%); however, the lower limit set on recording in the biosensor precludes further definitive understanding. The Abbott biosensor CGM used in this study has a lower detection limit of 54 mg/dL, making it unable to capture the full extent of LVL2 hypoglycemia. However, estimations of the time spent below 55 mg/dL [3.1 mM] were possible. Moreover, CGM accuracy tends to decrease during hypoglycaemia. In a study by Moser et al. (2019), the mean absolute relative difference (MARD) during the trial day was found to be 31.6% in hypoglycaemic conditions, compared to 16% during euglycaemia. Another potential influence is the occurrence of ‘compression lows,’ where pressure on the sensor causes falsely low readings (Mensh et al. 2013). Consequently, nighttime TBR readings should be interpreted with caution as readings could partly be due to body position during sleep.

24‐h time in range was somewhat different in our study compared to others which appear to report greater TIR or lesser TBR and TAR. Shah et al. (2019) used similar threshold limits over a 10‐d period reporting 24 h glycaemia as TBR 1.1%, TIR 96% and TAR 2.1%. Bowler et al. (2024) applied slightly different ranges: time below range (< 72 mg/dL), time in range (72–144 mg/dL) and time above range (> 144 mg/dL). They observed race walkers over a 4‐d period and reported 24 h glycaemia as TBR 0.5%, TIR 96.3% and TAR 2.4%. Skroce et al. (2024) retrospectively analysed the SuperSapiens user database which included 12,504 physically active individuals. They reported more similar time in ranges as TBR 3.4%, TAR 3.6% and the remainder within the target euglycaemic range (∼93%). Hamilton et al. (2024) observed 9 professional female cyclists during a 9‐day training camp and reported greater TBR with mean 24 h glycaemia as TBR 8%, TIR 93% and TAR 3%. Weijer et al. (2024) reported 24 h glycaemia as TBR 2.1, TIR 90.8% and TAR 5.4%. Taken together, along with our data and accepting there is currently no accepted threshold for hyperglycaemia, it would appear glycaemia is generally well controlled in healthy athletic individuals. Interestingly, in the study by T. Thomas et al. (2016), the researchers utilised an upper threshold of 126 mg/dL, which is lower than those used in the previously mentioned studies. This resulted in a higher proportion of time spent above range (TAR). Their findings highlight a need for the harmonisation of glycaemic thresholds for athletic individuals.

### 4.2 28‐Day Nutrition and Training Data

Diet logging by athletes was recorded as 97% for both low‐ and high‐GI carbohydrate diet arms, indicating a high adherence by study participants even though inaccurate reporting is a recognised limitation of the collection of diet logs (Burke et al. 2001). Daily energy intakes were isocaloric during both 28‐day diet arms. It is recognised that protein and fat content of foods can influence a food glycaemic index (Jenkins et al. 1981) and low‐GI foods can contain more protein and/or fat in comparison to high‐GI versions. There was a ∼14g.d^−1^ greater consumption of protein in the LGI carbohydrate diet than in the HGI carbohydrate arm. The mean CHO intakes per day in our cohort were 5.4 and 5.6 g.kg^−1^. d^−1^ for LGI and HGI carbohydrate diets respectively. These are lower than our initial suggestions. However, given the overall volume and intensity of the recorded training, these intakes do fall within an appropriate range for fuelling general endurance training (5–7 g.kg^−1^. d^−1^) (T. Thomas et al. 2016). CHO intake as a percentage of daily energy intake was slightly lower in the LGI than in the HGI diet arm (LGI 56 ± 3 vs. HGI 60 ± 3%, *p* < 0.001). This is likely to be explained by the slightly greater daily intake of fat (not statistically significant) and higher protein in the LGI diet. Nonetheless, the carbohydrate intakes meet the minimum recommendation of ∼ 45% of daily intake for active individuals (Manore 2005). It is worth noting that assigning strict ratio‐based intakes has been criticised for leading to unrealistic and unnecessary recommendations in some cases (Burke et al. 2001). Thus, an absolute and relativised gram‐to‐body mass approach is deemed more appropriate and less problematic when supporting highly active individuals (Burke et al. 2001; Manore 2005).

Training volumes were similar in both LGI and HGI carbohydrate diet arms and typical of competitive ultra‐runners. Volume may depend on the background of the athlete and the specific event. A weekly volume of ∼9 h is the reported average in the typical ultra‐marathoner completing multi‐day events (Rüst, Knechtle, Knechtle, et al. 2012). The weekly training volume in this study was ∼9.5 and ∼8.8 h for low‐ or high‐GI diet arms, respectively, a value that is lower in volume than might be observed in other ultra‐endurance sports such as Ironman Triathlon and ultra‐endurance cycling but might speak to the periodised training phase of the year. For comparison, reported training volumes for amateur Ironman triathletes are ∼14 and ∼12 h per week for ultra‐endurance cycling (Rüst et al. 2012).

### 4.3 Run Capacity

Before and after embarking on a 28‐day period of following an LGI or HGI carbohydrate diet, participants completed an acute laboratory run capacity trial to exhaustion (74 ± 1% v$\dot{V}$O_2peak_) following a 3‐h standardised run. There were no differences across all carbohydrate trials in endurance performance variables (Table 3) when refeeding with either isomaltulose or maltodextrin, nor was there a difference before or after 28 days on either LGI or HGI carbohydrate‐rich diets. Endurance running duration to volitional exhaustion was similar across trials with similar improvements after both periods. Physiological measures were similar before and after 28 days following both diet arms, although there was a small significant difference in the change in mean HR after each diet. This difference was greater in the HGI diet arm. As the CHO quantity was matched, both CHO sources were both likely to provide sufficient glucose to the working muscle, although through different pathways (Fuchs et al. 2019; Jeukendrup 2010). Adopting a low‐glycaemic index carbohydrate fuelling strategy pre‐exercise has been shown to help maintain glucose stability (Ching‐Lin et al. 2003; Thomas et al., 1994). However, improved glucose stability has not consistently been linked to improved performance outcomes (Burdon et al. 2017).

Carbohydrate oxidation with isomaltulose was less than that of maltodextrin before the 28‐day diets began. After 28 days of an LGI diet, carbohydrate oxidation was significantly lower under the isomaltulose arms when compared to the maltodextrin arm values. The lipid oxidation rate before engaging in a 28‐day diet was greater under isomaltulose than maltodextrin and increased to a greater extent after following a 28‐day LGI carbohydrate diet. This study reveals that the reduction in lipid oxidation typically seen after consuming carbohydrate was less pronounced with isomaltulose than with maltodextrin, both acutely and over a 28‐day period following an LGI diet. These results suggest that the benefit of minimised lipid oxidation suppression with isomaltulose persists with regular, prolonged consumption. This is often seen as a favourable adaptation due to the potential fuel stores in adipose tissue compared to relatively limited glycogen stores (Hawley et al. 1998). Although there is limited evidence to support increased fat oxidation improving endurance performance, some studies indicate it is beneficial, particularly in longer‐duration endurance events (Frandsen et al. 2017; Rowlands and Hopkins 2002).

### 4.4 Future Considerations

In this study, adopting a low‐glycaemic index diet reduced glycaemic variability and improved time spent in the euglycaemic range. The reduced time spent in hypoglycaemia may suggest that low glycaemic index carbohydrate sources may help maintain better glucose stability over longer periods than high‐GI carbohydrate diets. The long‐term downstream impact of this is less well‐known, although LGI diets have been linked with improved ratings of well‐being, mood and cognitive function in some studies (Phillippou and Constantinou 2014; Sünram‐Lea and Owen 2017), the proposed mechanism being more stable glycaemia throughout the course of a day. Cognitive performance and decision‐making are integral factors for an athlete to perform at their best. LGI diets appear to offer a supportive role in multiple facets to support athletes both in daily life and competition.

In addition to glycaemic control, improved lipid oxidation during exercise is a much‐desired adaptation for both athletes and coaches. Commonly applied methods often involve some form of carbohydrate restriction or manipulation, which runs the risk of hindering high‐intensity performance (Burke and Whitfield 2023; Burke et al. 2020; Impey et al. 2018). This can also quickly contribute to health issues like inadequate energy intake and relative energy deficiency in sport (RED‐S) (Burke 2010; Burke et al. 2001; Stellingwerff et al. 2021). The metabolic benefits observed in this study, while maintaining endurance capacity, may offer a more appealing strategy.

### 4.5 Strengths and Limitations

A key strength of this study was the reasonably long randomised observation period, which included two trial arms that collected glycaemic data, dietary information and physical activity data with strong adherence to logging under controlled conditions. However, extending the observation period may provide greater insights into the long‐term health implications of a high‐carbohydrate diet. This design also allowed for the assessment of adaptive responses, supported by controlled laboratory trials. However, the study was limited by a relatively small sample size, although appropriate for the scope of the observations. Additionally, there was less female representation than desired, preventing the assessment of biological sex as a potential variable (Cowley et al. 2021). In terms of the performance assessment, tests to exhaustion are associated with some limitations concerning reliability (Laursen et al. 2007). However, our findings were in line with other similar studies in the available literature (Burdon et al. 2017).

## 5 Conclusion

This study investigated the influence of a 28‐d low‐ and high‐glycaemic index carbohydrate diet on glycaemic control and the impact on endurance capacity in ultra‐endurance athletes. Continuous glucose monitoring revealed that a low‐glycaemic diet, incorporating isomaltulose, improved glycaemic stability, reduced time in hypoglycaemia and promoted lipid oxidation with similar endurance run capacity.

## Author Contributions

R.A.H., O.M.M., R.X., C.N., R.C. and R.M.B. contributed to the conception and design of the study. R.A.H., O.M.M., R.X. and C.N. were responsible for the acquisition of data. R.A.H. was responsible for the statistical analysis of the data. All authors were responsible for data interpretation. R.A.H., O.M.M. and R.M.B. co‐wrote the original draft of the manuscript. All authors contributed to revising the article. All authors provided final approval of the version to be published.

## Ethics Statement

Ethical approval was granted by the Swansea University Research Ethics Committee. The study was carried out in accordance with the Declaration of Helsinki and International Conference on Harmonisation Good Clinical Practice. Only data collected from cyclists that provided informed consent were included in the study.

## Conflicts of Interest

The study results and data contained in this publication have been developed by and/or for BENEO. BENEO reserves the exclusive right to use the results and data for possible Health Claims requests. Supersapiens had no part in the production, design or interpretation of data in this research study. The authors report no other conflicts of interest. The results of the study are presented clearly, honestly, and without fabrication, falsification, or inappropriate data manipulation.

## Supporting information


**Table S1:** Summary of [iG] metrics during each pre‐defined time‐period: rest, 3 h run, refeed, run test to exhaustion and recovery under each carbohydrate (isomaltulose [ISO] or maltodextrin [MAL]) condition both before (pre) and after (post) each 28‐day period.

## References

[ejsc70092-bib-0001] Anderson, J. , S. Attvall , L. Sternemalm , et al. 2011. “Effect on Glycemic Control by Short‐ and Long‐Term Use of Continuous Glucose Monitoring in Clinical Practice.” Journal of Diabetes Science and Technology 5, no. 6: 1472–1479. 10.1177/193229681100500622. 22226268 PMC3262717

[ejsc70092-bib-0002] BASES . 2006. Sport and Exercise Physiology Testing Guidelines: Volume I ‐ Sport Testing. Routledge.

[ejsc70092-bib-0003] Bazzano, L. , M. Serdula , and S. Liu . 2005. “Prevention of Type 2 Diabetes by Diet and Lifestyle Modification.” Journal of the American College of Nutrition 24, no. 5: 310–319. 10.1080/07315724.2005.10719479. 16192254

[ejsc70092-bib-0004] Bergia, R. , R. Giacco , T. Hjorth , et al. 2022. “Differential Glycemic Effects of Low‐ Versus High‐Glycemic Index Mediterranean‐Style Eating Patterns in Adults at Risk for Type 2 Diabetes: The MEDGI‐Carb Randomized Controlled Trial.” Nutrients 14, no. 3: 706. 10.3390/nu14030706. 35277067 PMC8838655

[ejsc70092-bib-0005] Bowler, A. , L. M. Burke , V. G. Coffey , and G. R. Cox . 2024. “Day‐To‐Day Glycemic Variability Using Continuous Glucose Monitors in Endurance Athletes.” Journal of Diabetes Science and Technology: 1–8. 10.1177/19322968241250355. PMC11572009 38726672

[ejsc70092-bib-0006] Brun, J. , M. Dumortier , C. Fedou , and J. Mercier . 2001. “Exercise Hypoglycemia in Nondiabetic Subjects.” Diabetes & Metabolism 27, no. 2 Pt. 1: 92–106. https://pubmed.ncbi.nlm.nih.gov/11353874/. 11353874

[ejsc70092-bib-0007] Burdon, C. , I. Spronk , H. L. Cheng , and H. T. O’Connor . 2017. “Effect of Glycemic Index of a Pre‐Exercise Meal on Endurance Exercise Performance: A Systematic Review and Meta‐Analysis.” Sports Medicine 47, no. 6: 1087–1101. 10.1007/s40279-016-0632-8. 27677914

[ejsc70092-bib-0008] Burke, L. 2010. “Fueling Strategies to Optimize Performance: Training High or Training Low?” Supplement, Scandinavian Journal of Medicine & Science in Sports 20, no. s2: 48–58. 10.1111/j.1600-0838.2010.01185.x. 20840562

[ejsc70092-bib-0009] Burke, L. , G. R. Collier , and M. Hargreaves . 1998. “Glycemic Index‐A New Tool in Sport Nutrition?” International Journal of Sport Nutrition 8, no. 4: 401–415. 10.1123/ijsn.8.4.401. 9841960

[ejsc70092-bib-0010] Burke, L. , G. R. Cox , N. K. Cummings , and B. Desbrow . 2001. “Guidelines for Daily Carbohydrate Intake. Do Athletes Achieve Them?” Sports Medicine 31, no. 4: 267–299. 10.2165/00007256-200131040-00003. 11310548

[ejsc70092-bib-0011] Burke, L. , and J. Whitfield . 2023. “Ketogenic Diets are Not Beneficial for Athletic Performance.” Medicine & Science in Sports & Exercise 56, no. 4: 756–759. 10.1249/mss.0000000000003344. 38079311

[ejsc70092-bib-0012] Burke, L. , J. Whitfield , I. A. Heikura , et al. 2020. “Adaptation to a Low Carbohydrate High Fat Diet is Rapid but Impairs Endurance Exercise Metabolism and Performance Despite Enhanced Glycogen Availability.” Journal of Physiology 599, no. 3: 727–728. 10.1113/jp280221. 32697366 PMC7891450

[ejsc70092-bib-0013] Chekima, K. , M. I. Noor , Y. B. H. Ooi , S. W. Yan , M. Jaweed , and B. Chekima . 2022. “Utilising a Real‐Time Continuous Glucose Monitor as Part of a Low Glycaemic Index and Load Diet and Determining Its Effect on Improving Dietary Intake, Body Composition and Metabolic Parameters of Overweight and Obese Young Adults: A Randomised Controlled Trial.” Foods 11, no. 12: 1754. 10.3390/foods11121754. 35741952 PMC9222336

[ejsc70092-bib-0014] Ching‐Lin, W. , C. Nicholas , C. Williams , A. Took , and L. Hardy . 2003. “The Influence of High‐Carbohydrate Meals With Different Glycaemic Indices on Substrate Utilisation During Subsequent Exercise.” British Journal of Nutrition 90, no. 6: 1049–1056. 10.1079/bjn20031006. 14641964

[ejsc70092-bib-0015] Cowley, E. , A. A. Olenick , K. L. McNulty , and E. Z. Ross . 2021. “Â“Invisible SportswomenÂ”: The Sex Data Gap in Sport and Exercise Science Research.” Women in Sport & Physical Activity Journal 29, no. 2: 146–151. 10.1123/wspaj.2021-0028.

[ejsc70092-bib-0016] Cryer, E. 2007. “Hypoglycemia, Functional Brain Failure, and Brain Death.” Journal of Clinical Investigation 117, no. 4: 868–870. 10.1172/jci31669. 17404614 PMC1838950

[ejsc70092-bib-0017] Danne, T. , R. Nimri , T. Battelino , et al. 2017. “International Consensus on Use of Continuous Glucose Monitoring.” Diabetes Care 40, no. 12: 1631–1640. 10.2337/dc17-1600. 29162583 PMC6467165

[ejsc70092-bib-0018] Flockhart, M. , and F. Larsen . 2023. “Continuous Glucose Monitoring in Endurance Athletes: Interpretation and Relevance of Measurements for Improving Performance and Health.” Sports Medicine 54, no. 2: 247–255. 10.1007/s40279-023-01910-4. 37658967 PMC10933193

[ejsc70092-bib-0019] Flockhart, M. , L. C. Nilsson , S. Tais , B. Ekblom , W. Apró , and F. J. Larsen . 2021. “Excessive Exercise Training Causes Mitochondrial Functional Impairment and Decreases Glucose Tolerance in Healthy Volunteers.” Cell Metabolism 33, no. 5: 957–970. 10.1016/j.cmet.2021.02.017. 33740420

[ejsc70092-bib-0020] Francois, M. , S. D. Cosgrove , N. M. Walker , S. J. E. Lucas , and K. E. Black . 2018. “Physiological Responses to a Five‐Day Adventure Race: Continuous Blood Glucose, Hemodynamics and Metabolites the 2012 GODZone field‐study.” Journal of Exercise Science & Fitness 16, no. 3: 78–82. 10.1016/j.jesf.2018.07.002. 30662498 PMC6323162

[ejsc70092-bib-0021] Frandsen, J. , S. Vest , S. Larsen , F. Dela , and J. Helge . 2017. “Maximal Fat Oxidation Is Related to Performance in an Ironman Triathlon.” International Journal of Sports Medicine 38, no. 13: 975–982. 10.1055/s-0043-117178. 29050040

[ejsc70092-bib-0022] Fuchs, J. , J. T. Gonzalez , and L. J. C. van Loon . 2019. “Fructose Co‐Ingestion to Increas Carbohydrate Availability in Athletes.” Journal of Physiology (London) 597, no. 14: 3549–3560. 10.1113/jp277116. 31166604 PMC6852172

[ejsc70092-bib-0023] Galindo, R. , and G. Aleppo . 2020. “Continuous Glucose Monitoring: The Achievement of 100 Years of Innovation in Diabetes Technology.” Diabetes Research and Clinical Practice 170: 108502. 10.1016/j.diabres.2020.108502. 33065179 PMC7736459

[ejsc70092-bib-0024] Gunther, S. , and H. Heymann . 1998. “Di‐ and Oligosaccharide Substrate Specificities and Subsite Binding Energies of Pig Intestinal Glucoamylase‐Maltase.” Archives of Biochemistry and Biophysics 354, no. 1: 111–116. 10.1006/abbi.1998.0684. 9633604

[ejsc70092-bib-0025] Hamilton, R. , O. M. McCarthy , S. C. Bain , and R. M. Bracken . 2024. “Continuous Measurement of Interstitial Glycaemia in Professional Female UCI World Tour Cyclists Undertaking a 9‐Day Cycle Training Camp.” European Journal of Sport Science 24, no. 11: 1573–1582. 10.1002/ejsc.12201. 39340462 PMC11534661

[ejsc70092-bib-0026] Hawley, J. , F. Brouns , and A. Jeukendrup . 1998. “Strategies to Enhance Fat Utilisation During Exercise.” Sports Medicine 25, no. 4: 241–257. 10.2165/00007256-199825040-00003. 9587182

[ejsc70092-bib-0027] Henry, C. , B. Kaur , R. Quek , and S. Camps . 2017. “A Low Glycaemic Index Diet Incorporating Isomaltulose is Associated With Lower Glycaemic Response and Variability, and Promotes Fat Oxidation in Asians.” Nutrients 9, no. 5: 473. 10.3390/nu9050473. 28486426 PMC5452203

[ejsc70092-bib-0028] Impey, S. , M. A. Hearris , K. M. Hammond , et al. 2018. “Fuel for the Work Required: A Theoretical Framework for Carbohydrate Periodization and the Glycogen Threshold Hypothesis.” Sports Medicine 48, no. 5: 1031–1048. 10.1007/s40279-018-0867-7. 29453741 PMC5889771

[ejsc70092-bib-0029] Ishihara, K. , N. Uchiyama , S. Kizaki , E. Mori , T. Nonaka , and H. Oneda . 2020. “Application of Continuous Glucose Monitoring for Assessment of Individual Carbohydrate Requirement During Ultramarathon Race.” Nutrients 12, no. 4: 1121. 10.3390/nu12041121. 32316458 PMC7230511

[ejsc70092-bib-0030] Jenkins, D. , T. M. Wolever , R. H. Taylor , et al. 1981. “Glycemic Index of Foods: A Physiological Basis for Carbohydrate Exchange.” American Journal of Clinical Nutrition 34, no. 3: 362–366. 10.1093/ajcn/34.3.362. 6259925

[ejsc70092-bib-0031] Jeukendrup, A. 2010. “Carbohydrate and Exercise Performance: The Role of Multiple Transportable Carbohydrates.” Current Opinion in Clinical Nutrition and Metabolic Care 13, no. 4: 452–457. 10.1097/mco.0b013e328339de9f. 20574242

[ejsc70092-bib-0032] Jeukendrup, A. , and S. Killer . 2010. “The Myths Surrounding Pre‐Exercise Carbohydrate Feeding.” Supplement, Annals of Nutrition and Metabolism 57, no. S2: 18–25. 10.1159/000322698. 21346333

[ejsc70092-bib-0033] Kaur, B. , R. Quek Yu Chin , S. Camps , and C. J. Henry . 2016. “The Impact of a Low Glycaemic Index (GI) Diet on Simultaneous Measurements of Blood Glucose and Fat Oxidation: A Whole Body Calorimetric Study.” Journal of Clinical & Translational Endocrinology 4: 45–52. 10.1016/j.jcte.2016.04.003. 29159130 PMC5680450

[ejsc70092-bib-0034] Keshet, A. , S. Shilo , A. Godneva , et al. 2023. “CGMAP: Characterising Continuous Glucose Monitor Data in Thousands of Non‐Diabetic Individuals.” Cell Metabolism 35, no. 5: 758–769. 10.1016/j.cmet.2023.04.002. 37080199

[ejsc70092-bib-0035] Kulawiec, D. , et al. 2021. “Continuous Glucose Monitoring to Measure Metabolic Impact and Recovery in Sub‐Elite Enduance Athletes.” Biomedical Signal Processing and Control 70. 10.1016/j.bspc.2021.103059.

[ejsc70092-bib-0036] Laursen, P. , G. T. Francis , C. R. Abbiss , M. J. Newton , and K. A. Z. U. N. O. R. I. Nosaka . 2007. “Reliability of Time‐to‐Exhaustion Versus Time‐Trial Running Tests in Runners.” Medicine & Science in Sports & Exercise 39, no. 8: 1374–1379. 10.1249/mss.0b013e31806010f5. 17762371

[ejsc70092-bib-0037] Lina, B. , D. Jonker , and G. Kozianowski . 2002. “Isomaltulose (Palatinose1): A Review of Biological and Toxicological Studies.” Food and Chemical Toxicology 40, no. 10: 1375–1381. 10.1016/s0278-6915(02)00105-9. 12387299

[ejsc70092-bib-0038] Manore, M. 2005. “Exercise and the Institute of Medicine Recommendations for Nutrition.” Current Sports Medicine Reports 4: 193–198. 10.1097/01.csmr.0000306206.72186.00. 16004827

[ejsc70092-bib-0039] Maresch, C. , S. F. Petry , S. Theis , A. Bosy‐Westphal , and T. Linn . 2017. “Low Glycemic Index Prototype Isomaltulose—Update of Clinical Trials.” Nutrients 9, no. 381: 381. 10.3390/nu9040381. 28406437 PMC5409720

[ejsc70092-bib-0040] Mensh, B. , N. A. Wisniewski , B. M. Neil , and D. R. Burnett . 2013. “Susceptibility of Interstitial Continuous Glucose Monitor Performance to Sleeping Position.” Journal of Diabetes Science and Technology 7, no. 4: 863–870. 10.1177/193229681300700408. 23911167 PMC3879750

[ejsc70092-bib-0041] Moore, L. , A. W. Midgley , G. Thomas , S. Thurlow , and L. R. McNaughton . 2009. “The Effects of Low– and High–Glycemic Index Meals on Time Trial Performance.” International Journal of Sports Physiology and Performance 4, no. 3: 331–344. 10.1123/ijspp.4.3.331. 19953821

[ejsc70092-bib-0042] Moore, L. , A. W. Midgley , S. Thurlow , G. Thomas , and L. R. Mc Naughton . 2010. “Effect of the Glycaemic Index of a Pre‐Exercise Meal on Metabolism and Cycling Time Trial Performance.” Journal of Science and Medicine in Sport 13, no. 1: 182–188. 10.1016/j.jsams.2008.11.006. 19230767

[ejsc70092-bib-0043] Moser, O. , M. L. Eckstein , O. McCarthy , et al. 2019. “Performance of the Freestyle Libre Flash Glucose Monitoring (Flash GM) System in Individuals With Type 1 Diabetes: A Secondary Outcome Analysis of a Randomized Crossover Trial.” Diabetes, Obesity and Metabolism 21, no. 11: 2505–2512. 10.1111/dom.13835. PMC6852439 31332929

[ejsc70092-bib-0044] Notbohm, H. , J. F. Feuerbacher , F. Papendorf , et al. 2021. “Metabolic, Hormonal and Performance Effects of Isomaltulose Ingestion Before Prolonged Aerobic Exercise: A double‐blind, Randomised, Cross‐Over Trial.” Journal of The International Society of Sports Nutrition 18, no. 38: 38. 10.1186/s12970-021-00439-z. 34001166 PMC8130436

[ejsc70092-bib-0045] Phillippou, E. , and M. Constantinou . 2014. “The Influence of Glycemic Index on Cognitive Functioning: A Systematic Review of the Evidence.” American Society for Nutrition 5, no. 2: 119–130. 10.3945/an.113.004960. PMC3951795 24618754

[ejsc70092-bib-0046] Prins, P. , T. D. Noakes , A. Buga , et al. 2023. “Low and High Carbohydrate Isocaloric Diets on Performance, Fat Oxidation, Glucose and Cardiometabolic Health in Middle Age Males.” Frontiers in Clinical Nutrition 10: 1084021. 10.3389/fnut.2023.1084021. PMC9946985 36845048

[ejsc70092-bib-0047] Rowlands, D. , and W. Hopkins . 2002. “Effects of High‐Fat and High‐Carbohydrate Diets on Metabolism and Performance in Cycling.” Metabolism 51, no. 6: 678–690. 10.1053/meta.2002.32723. 12037719

[ejsc70092-bib-0048] Rüst, C. , B. Knechtle , P. Knechtle , and T. Rosemann . 2012. “Similarities and Differences in Anthropometry and Training Between Recreational Male 100‐km Ultra‐Marathoners and Marathoners.” Journal of Sports Sciences 30, no. 12: 1249–1257. 10.1080/02640414.2012.697182. 22724447

[ejsc70092-bib-0049] Seiler, S. 2010. “What is Best Practice for Training Intensity and Duration Distribution in Endurance Athletes.” International Journal of Sports Physiology and Performance 5, no. 3: 276–291. 10.1123/ijspp.5.3.276. 20861519

[ejsc70092-bib-0050] Sengoku, Y. , K. Nakamura , H. Ogata , Y. Nabekura , S. Nagasaka , and K. Tokuyama . 2015. “Continuous Glucose Monitoring During a 100‐km Race: A Case Study in an Elite Ultramarathon Runner.” International Journal of Sports Physiology and Performance 10, no. 1: 124–127. 10.1123/ijspp.2013-0493. 24896042

[ejsc70092-bib-0051] Shah, V. , S. N. DuBose , Z. Li , et al. 2019. “Continuous Glucose Monitoring Profiles in Healthy Nondiabteic Participants: A Multicenter Prospective Study.” Journal of Clinical Endocrinology and Metabolism 104, no. 10: 4356–4364. 10.1210/jc.2018-02763. 31127824 PMC7296129

[ejsc70092-bib-0052] Simpson, E. , M. Holdsworth , and I. A. Macdonald . 2008. “Interstitial Glucose Profile Associated With Symptoms Attributed to Hypoglycemia by Otherwise Healthy Women.” American Journal of Clinical Nutrition 87, no. 2: 354–361. 10.1093/ajcn/87.2.354. 18258625

[ejsc70092-bib-0053] Skroce, K. , A. Zignoli , F. Y. Fontana , et al. 2024. “Real World Interstitial Glucose Profiles of a Large Cohort of Physically Active Men and Women.” Sensors 24, no. 3: 744. 10.3390/s24030744. 38339464 PMC10857405

[ejsc70092-bib-0054] Stellingwerff, T. 2016. “Competition Nutrition Practices of Elite Ultramarathon Runners.” International Journal of Sport Nutrition and Exercise Metabolism 26, no. 1: 93–99. 10.1123/ijsnem.2015-0030. 26061831

[ejsc70092-bib-0055] Stellingwerff, T. , I. A. Heikura , R. Meeusen , et al. 2021. “Overtraining Syndrome (OTS) and Relative Energy Deficiency in Sport (RED‐S): Shared Pathways, Symptoms and Complexities.” Sports Medicine 51, no. 11: 2251–2280. 10.1007/s40279-021-01491-0. 34181189

[ejsc70092-bib-0056] Sünram‐Lea, S. , and L. Owen . 2017. “The Impact of Diet‐Based Glycaemic Response and Glucose Regulation on Cognition: Evidence Across the Lifespan.” Proceedings of the Nutrition Society 76, no. 4: 466–477. 10.1017/s0029665117000829. 28651658

[ejsc70092-bib-0057] Tanda, G. , and B. Knechtle . 2015. “Effects of Training and Anthropometric Factors on Marathon and 100 Km Ultramarathon Race Performance.” Open Access Journal of Sports Medicine 6: 129–136. 10.2147/oajsm.s80637. 25995653 PMC4425319

[ejsc70092-bib-0058] Thomas, D. , J. Brotherhood , and J. Brand . 1991. “Carbohydrate Feeding Before Exercise: Effect of Glycaemic Index.” International Journal of Sports Medicine 12, no. 2: 180–186. 10.1055/s-2007-1024664. 1860741

[ejsc70092-bib-0059] Thomas, D. , J. Brotherhood , and J. Brand . 1994. “Plasma Glucose Levels After Prolonged Strenuous Exercise Correlate Inversely With Glycemic Response to Food Consumed Before Exercise.” International Journal of Sport Nutrition, 4, no. 4: 361–373. 10.1123/ijsn.4.4.361. 7874152

[ejsc70092-bib-0060] Thomas, T. , K. A. Erdman , and L. M. Burke . 2016. “American College of Sports Medicine Joint Position Statement. Nutrition and Athletic Performance.” Medicine & Science in Sports & Exercise 48, no. 3: 543–568. 10.1249/MSS.0000000000000852. 26891166

[ejsc70092-bib-0061] van Can, J. , T. H. Ijzerman , L. J. C. van Loon , F. Brouns , and E. E. Blaak . 2009. “Reduced Glycaemic and Insulinaemic Responses Following Isomaltulose Ingestion: Implications for Postprandial Substrate Use.” British Journal of Nutrition 102, no. 10: 1408–1413. 10.1017/s0007114509990687. 19671200

[ejsc70092-bib-0062] Weijer, V. , R. van der Werf , M. van der Haijden , A. Jeukendrup , L. J. C. van Loon , and J.‐W. van Dijk . 2024. “Continuous Glucose Monitoring in Para Cyclists: An Observational Study.” Journal of Sports Sciences 24, no. 12: 1809–1819. 10.1002/ejsc.12220. PMC11621371 39587809

[ejsc70092-bib-0063] Wong, S. , P. M. Siu , A. Lok , Y. J. Chen , J. Morris , and C. W. Lam . 2008. “Effect of the Glycaemic Index of Pre‐Exercise Carbohydrate Meals on Running Performance.” European Journal of Sport Science 8, no. 1: 23–33. 10.1080/17461390701819451.

[ejsc70092-bib-0064] Wu, C. , and C. Williams . 2006. “A Low Glycemic Index Meal Before Exercise Improves Endurance Running Capacity in Men.” International Journal of Sport Nutrition and Exercise Metabolism 16, no. 5: 510–527. 10.1123/ijsnem.16.5.510. 17240783

[ejsc70092-bib-0065] Zignoli, A. , F. Y. Fontana , D. J. Lipman , K. Skroce , F. M. Maturana , and H. C. Zisser . 2023. “Association Between Pre‐Exercise Food Ingestion Timing and Reactive Hypoglycemia: Insights From a Large Database of Continuous Glucose Monitoring Data.” European Journal of Sport Science 23, no. 12: 2340–2348. 10.1080/17461391.2023.2233468. 37424300

[ejsc70092-bib-0066] Zignoli, A. , B. Martinez‐Gonzalez , K. Skroce , D. J. Lipman , H. C. Zisser , and A. Giorgi . 2024. “Minimum Overnight Interstitial Glucose Concentration in Professional Cyclists During Two Consecutive Annual Training Camps: The Limited Impact of Daily Exercise Energy Expenditure.” International Journal of Sport Nutrition and Exercise Metabolism 35, no. 3: 243–254. 10.1123/ijsnem.2024-0119. 39662483

