# Supplementary material for: Glycaemic Impact of Low‐ and High‐Glycaemic Index Carbohydrate Diets in Ultra‐Endurance Athletes: Insights From Continuous Glucose Monitoring
Source: Eur J Sport Sci. 2025 Nov 25;25(12):e70092. doi: 10.1002/ejsc.70092 (PMC12958867; doi:10.1002/ejsc.70092)
Supplement: Supplementary file 1 — Table S1: Summary of [iG] metrics during each pre‐defined time‐period: rest, 3 h run, refeed, run test to exhaustion and recovery under each carbohydrate (isomaltulose [ISO] or maltodextrin [MAL]) condition both before (pre) and after (post) each 28‐day period. [file EJSC-25-e70092-s001.docx]

**Supplemental Table 1:**

|  |  | ISO _pre-diet_ | ISO _post-diet_ | MAL _pre-diet_ | MAL _post-diet_ | *p value* | Δ LGI | Δ HGI |
| --- | --- | --- | --- | --- | --- | --- | --- | --- |
| Rest | Max (mg/dL) | 101.0 ± 23.7 | 113.3 ± 15.8 | 115.2 ± 23.2 | 103.8 ± 10.6 | *0.339* | +9.0 ± 36.0 | -10.5 ± 26.8 |
|  | Mean (mg/dL) | 93.1 ± 20.2 | 104.9 ± 11.6 | 97.0 ± 18.7 | 94.6 ± 9.3 | *0.444* | +8.4 ± 26.5 | -2.5 ± 22.0 |
|  | Min (mg/dL) | 86.6 ± 18.6 | 97.8 ± 10.3 | 86.6 ± 17.3 | 86.0 ± 11.5 | *0.331* | +7.3 ± 22.4 | -1.8 ± 25.1 |
|  | SD (mg/dL) | 4.8 ± 3.4 | 5.0 ± 2.4 | 7.3 ± 3.8 | 5.8 ± 2.5 | *0.349* | +0.4 ± 5.3 | -0.4 ± 4.0 |
|  | CV % | 5.0 ± 3.4 | 5 ± 2 | 8 ± 5 | 6 ± 3 | *0.241* | +0 ± 4 | +0 ± 5 |
|  | TAR % | 0 ± 0 | 1 ± 2 | 0 ± 0 | 0 ± 0 | *0.349* | +1 ± 2 | +0 ± 0 |
|  | TIR % | 80 ± 40 | 99 ± 2 | 89 ± 20 | 100 ± 0 | *0.223* | +12 ± 33 | +9 ± 20 |
|  | TBR % | 20 ± 40 | 0 ± 0 | 12 ± 20 | 0 ± 0 | *0.209* | -13 ± 36 | -9 ± 20 |
| 3H Run | Max mg/dL | 134.4 ± 18.4 | 139.0 ± 19.0 | 141.7 ± 9.9 | 137.1 ± 13.1 | *0.787* | 5.3 ± 26.3 | -7.3 ± 12.2 |
|  | Mean (mg/dL) | 111.2 ± 12.3 | 107.6 ± 13.8 | 109.9 ± 12.2 | 106.9 ± 10.0 | *0.866* | -5.8 ± 19.6 | -4.1 ± 17.0 |
|  | Min mg/dL | 92.7 ± 13.2 | 82.0 ± 17.0 | 86.8 ± 16.0 | 82.9 ± 12.2 | *0.425* | -14.5 ± 17.8 | +0.9 ± 15.5 |
|  | SD (mg/dL) | 10.8 ± 6.3 | 11.6 ± 3.0 | 12.6 ± 3.7 | 12.3 ± 7.6 | *0.891* | **+1.8 ± 4.1** | **-3.0 ± 2.4** |
|  | CV % | 10 ± 6 | 11 ± 3 | 12 ± 4 | 11.3 ± 6.3 | *0.845* | +2 ± 3 | -3 ± 4 |
|  | TAR % | 3 ± 10 | 3 ± 5 | 4 ± 5 | 3 ± 4 | *0.985* | -1 ± 13 | -1 ± 7 |
|  | TIR % | 96 ± 10 | 96 ± 4 | 94 ± 8 | 95 ± 8 | *0.925* | 0 ± 12 | +4 ± 8 |
|  | TBR % | 1 ± 3 | 1 ± 2 | 3 ± 8 | 3 ± 7 | *0.880* | +1 ± 2 | -3 ± 8 |
| Refeed period | Max mg/dL | 132.9 ± 15.6 | 142.8 ± 13.4 | 171.0 ± 27.8^†^ | 168.4 ± 27.1 | ***0.002*** | +10.4 ± 17.4 | +8.1 ± 18.7 |
|  | Mean (mg/dL) | 104.5 ± 12.4 | 110.2 ± 10.2 | 122.0 ± 22.4 | 115.2 ±13.5 | *0.131* | +3.1 ± 11.1 | -1.6 ± 15.0 |
|  | Min mg/dL | 74.6 ± 10.6 | 72.8 ± 13.3 | 73.9 ± 14.1 | 64.9 ± 9.1 | *0.300* | -9.0 ± 25.4 | -8.8 ± 17.9 |
|  | SD (mg/dL) | 12.2 ± 3.8 | 16.3 ± 4.9 | 27.2 ± 10.4^†^ | 23.7 ± 8.0 | ***0.001*** | +5.4 ± 4.4 | +0.8 ± 9.7 |
|  | CV % | 12 ± 5 | 15 ± 4 | 23 ± 8^†^ | 20 ± 6 | ***0.002*** | **+5 ± 4** | **0 ± 10** |
|  | TAR % | 1 ± 1 | 8 ± 8 | 33 ± 22 | 18 ± 19 | ***0.001*** | +8 ± 7 | -10 ± 17 |
|  | TIR % | 95 ± 13 | 91 ± 8 | 59 ± 16 | 79 ± 18 | ***<0.001*** | **-9 ± 7** | **+18 ± 23** |
|  | TBR % | 5 ± 13.3 | 1 ± 1 | 8 ± 18 | 4 ± 8 | *0.709* | +1 ± 2 | -8 ± 20 |
| Run test | Max mg/dL | 111.6 ± 25.3 | 122.0 ± 18.3 | 116.6 ± 32.1 | 114.9 ± 16.1 | *0.844* | +2.4 ± 30.0 | +11.6 ± 32.4 |
|  | Mean (mg/dL) | 94.6 ± 19.7 | 104.1 ± 14.1 | 88.4 ± 21.7 | 82.8 ± 10.4 | *0.098* | +5.7 ± 25.4 | +3.4 ± 24.8 |
|  | Min mg/dL | 79.1 ± 19.1 | 87.9 ± 16.3 | 62.4 ± 12.4 | 64.0 ± 19.8 | *0.767* | +21.7 ± 34.9 | +6.2 ± 29.4 |
|  | SD (mg/dL) | 10.2 ± 6.3 | 10.4 ± 7.1 | 17.8 ± 10.2 | 18.4 ± 5.0 | ***0.047*** | -1.8 ± 3.4 | +1.5 ± 10.0 |
|  | CV % | 11 ± 7 | 10 ± 8 | 19 ± 11 | 22 ± 5^#^ | ***0.008*** | -2 ± 3 | +6 ± 8 |
|  | TAR % | 0 ± 0 | 3 ± 7 | 7 ± 18 | 0 ± 0 | *0.382* | +3 ± 7 | -9 ± 20 |
|  | TIR % | 84 ± 35 | 93 ± 12 | 60 ± 32 | 69 ± 20 | *0.094* | -7 ± 9 | +14 ± 52 |
|  | TBR % | 16 ± 35 | 4 ± 11 | 33 ± 35 | 31 ± 20 | *0.146* | +3 ± 6 | -13 ± 52 |
| RECOVERY | Max mg/dL | 132.9 ± 20.0 | 133.1 ± 18.2 | 143.4 ± 36.6 | 143.7 ± 14.0 | *0.683* | -3.6 ± 23.8 | +0.2 ± 27.6 |
|  | Mean (mg/dL) | 101.2 ± 16.6 | 102.3 ± 8.2 | 100.7 ± 23.2 | 103.4 ± 8.7 | *0.987* | -4.2 ± 9.1 | +2.6 ± 21.2 |
|  | Min mg/dL | 79.8 ± 12.0 | 74.4 ± 9.0 | 70.3 ± 17.6 | 67.1 ± 7.7 | *0.230* | -9.6 ± 10.6 | -2.0 ± 17.0 |
|  | SD (mg/dL) | 11.6 ± 1.9 | 14.7 ± 5.4 | 18.0 ± 5.6 | 19.5 ± 4.3 | ***0.010*** | +3.8 ± 5.0 | +0.1 ± 4.9 |
|  | CV % | 12 ± 3 | 14 ± 5 | 18 ± 3^†^ | 19 ± 4 | ***0.002*** | +4 ± 4 | +0 ± 3 |
|  | TAR % | 0 ± 1 | 4 ± 8 | 10 ± 12 | 5 ± 9 | *0.182* | +3 ± 9 | -6 ± 9 |
|  | TIR % | 91 ± 25 | 95 ± 8 | 76 ± 14 | 89 ± 11 | *0.148* | -6 ± 10 | +15 ± 18 |
|  | TBR % | 9 ± 25 | 1 ± 3 | 14 ± 20 | 6 ± 9 | *0.503* | +2 ± 4 | -8 ± 20 |

**Supplemental Table 1:** Summary of [iG] metrics during each pre-defined time-period: rest, 3 h run, refeed, run test to exhaustion and recovery under each carbohydrate (isomaltulose [ISO] or maltodextrin [MAL]) condition both before (pre) and after (post) each 28-day period. Data expressed as mean ± SD (n=9). † indicates a difference between ISO and MAL arms before starting the 28-d diet. # indicates a difference between ISO and MAL arms after the 28-d diet (p≤0.05). Δ denotes the change within each of the dietary arms. Data expressed as mean ± SD (n=9). (p≤0.05).
